# Plant Microbiome Innovation: M-trophs

**DOI:** 10.1089/ind.2018.29131.tla

**Published:** 2018-06-01

**Authors:** Thomas Laurita, Janne Kerovuo

**Affiliations:** NewLeaf Symbiotics, St. Louis, MO

## Introduction

Human population is increasing from 7.6 billion today to an estimated 9.5–10 billion by 2050.^1^ How can billions more people, with increasing demands for protein and other food sources, be adequately fed without destroying the environment? Utilizing more land for agricultural purposes is not a solution, given that much of the arable land on the planet is already being used for food, fiber, chemical, or fuel production.^2^ One answer to this complex challenge will lie in the ability to produce more food within the current footprint in sustainable and affordable ways.

By one definition, “an agricultural biologicals system is an ecologically, socially, and economically sustainable agricultural production system that promotes safe products by minimizing environmental adverse consequences and reducing the use of non-renewable natural resources.”^3^ In this article, we discuss opportunities that capitalize on twenty-first century technological innovation and the central role played by the microbial life intimately associated with plants: the plant and soil microbiomes. We examine the potential of Ag biologicals within the agricultural ecosystem. We elucidate their functionality as a means of increasing plant health and crop yields while maintaining environmental sustainability. Finally we illustrate the role that NewLeaf Symbiotics (St. Louis, MO) plays in the introduction of a range of Ag biologicals based on methylobacteria, or M-trophs—bio-complementary products that infuse plants with microbes that are tailored to fit specific crop genetics and environmental conditions.^4^

Most major agricultural input suppliers and seed companies recognize that biologicals represent a largely untapped opportunity to meet demand for novel and sustainable complements to the existing roster of agricultural chemicals, fertilizers, and genetically modified (GM) traits. Recent government, industrial, and private investments support the belief that significant improvements in crop productivity and environmental sustainability are achievable using complementary biological inputs. Between 2012 and 2015, over $2 billion was invested by Ag multinationals in agreements, mergers, and the acquisition of small biologicals companies.^5^ These investments are driving the development of the next generation of Ag biologicals.

The broad recognition of the agricultural significance of the plant and soil microbiome is supported in large part by the reduced cost, increased speed, and improved accuracy of genomic sequencing. Simultaneous increases in computing power and analytics are driving the rapid evolution of new molecular biology tools directed at solving previously intractable problems. The recognition of the phyto-microbiome as a second genome, with the potential to supplement and interact with the plant genome, offers significant opportunity to improve crop health and yield.^6^ The persistent evolution of pest resistance to chemical pesticides and traits, combined with consumer demand for sustainable farming and food traceability, have created a strong and growing interest in new Ag biologicals.^7^ In the US there are 350-plus registered biocontrol agents based on 50-plus species. The market is growing at an average compounded annual growth rate (CAGR) greater than 14%. Every major international and domestic supplier of agronomic inputs is investing in this area.

Since most Ag biological products are based on naturally occurring microbes, consumer health and safety concerns about these products are reasonably mitigated. Growers are aware and ready to try new biologicals, in part because biological products like rhizobia and *Bacillus thuringiensis* (*Bt*) have been used and accepted for decades. There are, however, significant obstacles to the introduction of novel and efficacious Ag biologicals. Microbes are part of extremely complex soil and plant systems; discovery and delivery of effective products are no simple tasks. Historically, some Ag biological market offerings failed to deliver consistent efficacy equal to conventional inputs, causing the industry to regard Ag biologicals with some caution. Improved strains, advanced formulations, and recognition of the unique value Ag biologicals can provide has led to an increase in their global market share over the past decade from less than 1% to more than 5%. Two of the major strategic challenges to increased use of Ag biologicals are:

▪ Optimization of discovery, screening, and derivation of candidate microbes, and
▪ Alignment of product development with technical and economic drivers of contemporary production farming.

## Optimization

There exist hundreds of potential candidate genera of soil- and plant-associated microbes, many economically important crop species, wide variations in relevant environmental conditions and farming practices, large numbers of ever-evolving crop pests, and complex biological interconnectivity among all these factors. This multitude of interconnected variables creates an intractably large number of possible combinations. Some companies try to address this challenge through the deployment of mass in silico, in vitro, and/or controlled environment screening of microbes for growth promotion or activity against biotic or abiotic stressors. Large companies with the requisite resources instead go directly to the field, measuring yield from a broad swath of microbes applied to key crops. Most try to optimize a combination of the two approaches.

## Alignment

The development, manufacture, and sale of agricultural inputs like seeds, pesticides, fertilizers, and traits is highly concentrated among a small number of large corporations. Companies developing Ag biologicals must design R&D, production, and commercialization strategies that align their product offerings with the realities of the market. Start-up companies oftentimes emerge from promising early stage science, yet the discipline of a market-oriented mindset must be incorporated in a company's strategy from the outset. Most Ag biological startup companies have incorporated executive, management, and scientific leadership with history and perspective coming from decades of agricultural industry experience. This wealth of insight is accelerating the deployment of novel Ag biologicals.

## The Previous Generation

Currently the two most widely used types of Ag biologicals are rhizobia and bacillus-based products. Rhizobia are naturally occurring soil bacteria that fix atmospheric nitrogen after becoming established in root nodules of legumes. In North and South America, tens of millions of acres of soybean and other legumes (including peanuts, peas, and lentils) are inoculated with rhizobia as a seed treatment to increase soil nitrogen availability and yield. *Bt* was discovered in the early twentieth century and was first applied as a biological insecticide shortly thereafter.^8^ *Bt* applied as a foliar spray on high value crops provides an alternative to synthetic chemical treatments and can play a role in the management of residue levels and in the mitigation of resistance. Since 1996, plants have been modified to include *Bt* genes. Genetically modified crops including soybean, corn, cotton, and others produce proteins that are toxic to a variety of insects. In the US in 2017, 80% of cotton and 77% of corn planted contained *Bt* genes. Other *Bacillus*-based products are used to combat plant pathogens (*Bacillus subtilis*, *Bacillus firmus*, *Bacillus amyloliquefaciens*, *Bacillus pumilus*, etc.). Some of these products are stand-alone, while most are used in combination with existing insecticides or fungicides. Some are delivered as foliar applications while others are directed to the rhizosphere as seed treatments.

## Next-Generation Ag Biologicals

NewLeaf Symbiotics operates on the premise that the next generation of Ag biologicals will be designed and delivered as bio-complements to existing chemical and/or GM technologies. NewLeaf combines cutting edge early screening techniques with comparative genomics approaches that use genotype to phenotype associations to drive product development. The company works exclusively with one class of microbial organisms: M-trophs.

**Fig. 1.**
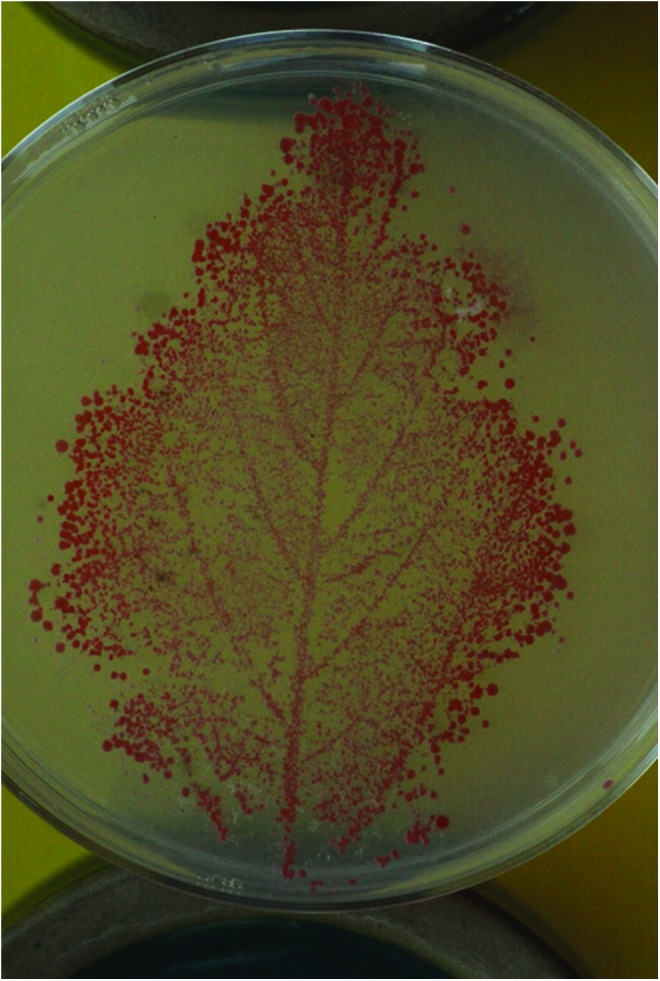
Soy bean leaf imprint on M-troph selective medium that precludes the rapid growth of the most commonly found commensal bacteria. The colonies are pink due to the presence of carotenoids expressed by M-trophs that serve as carbohydrate reserves and protect M-trophs from UV on the leaf surface.^16^ Color images available online at www.liebertpub.com/ind

Pink Pigmented Facultative Methylotrophs, or M-trophs for short, are robust and ubiquitous colonizing symbionts of most, if not all plants. Their symbiotic nature, close association with plants and many beneficial effects are well-suited to applications in sustainable agriculture.^9^ M-trophs can utilize C1 compounds such as methanol for biomass and energy. This gives them an advantage in plant colonization, since methanol is produced during plant growth due to the de-methylation of cell wall pectin by plant pectin methylesterases.^10^ The global and ubiquitous plant-associated nature of M-trophs was established in the pioneering work of Bill Corpe, who isolated M-trophs from 70 plant species.^11^ Another M-troph research pioneer, Mark Holland from Salisbury University, MD, has consistently isolated M-trophs from plant material of any plant species. He routinely measures 10e5–10e7 colony forming units (CFUs) of M-trophs per gram of fresh weight of leaf.^12^ It is believed that M-trophs have been co-evolving with plants perhaps even before plant cells evolved into plants, as some M-trophs promote the growth of algae,^13^ lichens, moss, and early bryophtes.^14,15^ *Figure 1* demonstrates the robust natural colonization of a soybean leaf by M-trophs.^16^

Not only are M-trophs ubiquitous plant colonizers, but they can constitute a large portion of the total bacterial population in the plant microbiome. Hirano and Upper reported that M-trophs constitute up to 79% of heterotrophic bacteria recovered from White Clover leaves and more than 90% of cultivable bacteria from Snap Bean.^17^ Using next-generation sequencing methods and bioinformatics, NewLeaf has demonstrated that M-trophs frequently constitute over 30% of the bacterial microbiome in soybean leaves in agricultural settings.

There are over 50 *Methylobacterium* species described in the literature. This reflects the interest among researchers about this particularly promising taxon of bacteria. Various M-trophs can live with the plant as epiphytes and endophytes, colonizing the phylloplane, rhizosphere, and endosphere.^9,18^ An intracellular Scots Pine shoot symbiont M-troph was recently discovered and demonstrated to aggregate around the host nucleus.^19^ This intracellular endophyte contained numerous eukaryotic-like protein domains indicating potential direct modulation of host plant gene expression.

M-trophs contain numerous known plant enhancing traits. Certain M-troph species can nodulate and fix nitrogen for the host plant.^20–22^ Many M-trophs secrete plant hormones such as gibberellin, cytokine, and auxin for the host plant, and modulate ethylene levels.^23,24^ Some M-trophs can protect the host plant by inducing systemic resistance while others produce antimicrobial compounds.^25–27^ M-trophs can also protect the host plant against pathogen attack by inducing endophyte community changes correlating with resistance to the disease.^28^ M-troph driven plant growth promotion has been demonstrated in numerous crops contributing to plant health as “Plant Growth-Promoting Rhizobacteria, PGRP.”^29^ Some M-trophs solubilize bound soil phosphate,^30^ while other M-trophs can be seed transmitted under the seed-coat and play an important role in seed germination.^31^

NewLeaf Symbiotics studies show that some M-troph strains increase the chlorophyll content and photosynthetic capacity of target crops when combined with certain agricultural inputs (data not shown). M-trophs likely play an important role in indirect and direct modulation of host plant gene expression and are true probiotics. M-trophs are generally desiccation tolerant. Desiccation tolerance is desirable for production of microbial products where a rapidly soluble powder formulation is acceptable. Other M-troph strains persist in a low water activity state and are therefore compatible with conventional emulsions and suspensions used for seed treatment. Many M-trophs contain hopanoid and trehalose biosynthesis genes which are both known to contribute to desiccation tolerance.^18^ Experiments on M-trophs mixed with commonly used agricultural chemistries (insecticides, fungicides, herbicides) demonstrate very high tolerance and compatibility.

The combination of numerous plant enhancing traits, a broadly probiotic nature, good desiccation tolerance and Ag chemistry compatibility, high achievable production titers, and low application dose rates, make M-trophs very attractive microbial tools for sustainable agriculture. Unlike some microbial products for agriculture, M-trophs are biosafety level one organisms. There is no evidence of M-troph pathogenicity to plants.

## New Class of Ag Biologicals

NewLeaf Symbiotics is solely focused on discovery and development of best fit M-trophs for a given product concept and crop. NewLeaf Symbiotics views M-trophs as an important new tool, part of a new class of products that bridge the gap between Ag biologicals, traditional chemistries, and/or GM traits.

The best fit M-trophs serve as drop-in solutions for agriculture. NewLeaf applies proprietary enrichments and isolation methods to target beneficial plant-associated M-trophs from samples collected from different biotopes around the continental US. NewLeaf collects principally from wild grass and legume crops and performs M-troph isolations from the phyllosphere, rhizosphere, and endosphere. All isolated strains go through whole genome sequencing followed by assembly and annotation using a proprietary computational bioinformatics engine, the Prescriptive Biologics Knowledgebase^™^.

The general beneficial, probiotic nature of M-trophs allows NewLeaf to nominate strains for field trials based on maximized genomic diversity and compatibility with commonly used Ag chemistries. Early plant colonization assays identify those strains that most efficiently colonize each target. NewLeaf also studies the fermentation and formulation profiles of candidate strains to vet for eventual large-scale production. This unorthodox approach to R&D, field trialing, and production has proven fruitful in creating a robust product pipeline, and in essence, a new class of Ag biologicals.

## Phenotype x Genotype Association Trials

In 2017, NewLeaf conducted a direct-to-field soybean yield trial. Elite soybean varieties coated with standard seed applied insecticides and fungicides (SAI/SAF) were treated with 67 genetically diverse M-troph cultures. M-trophs were selected using the Prescriptive Biologics Knowledge Base (PBK) bioinformatics tool to ensure a maximal M-troph genotypic diversity in the trial.

**Fig. 2.**
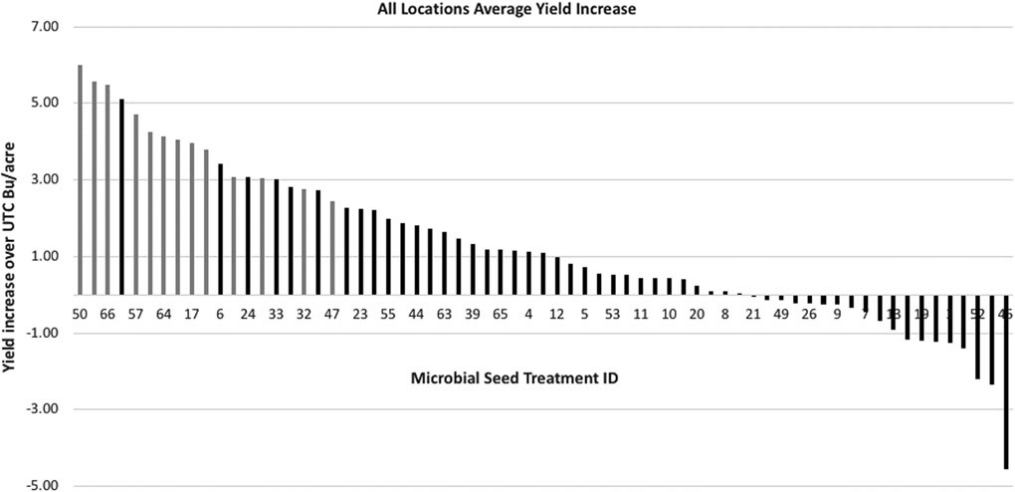
M-troph soy bean seed treatment yield performance. Best M-troph provided average of 6 bushel yield increase across all locations and increased early stand counts (data not shown). Top five M-troph treatments are statistically significantly different from the control. Gray bars indicate M-troph treatments that outperformed the untreated control in all 4 locations. These small plot trials with six replications per treatment were performed in Wisconsin, South Dakota, Indiana, and Missouri using elite soybean germplasm, standard agricultural practices, and chemical treatments.

Average yield increase versus control (SAI/SAF seed treatment only) across all locations is shown in *Fig. 2*. The best performing M-trophs delivered up to 6 bushel per acre yield increase with 100% win rate (yield increase versus control across all locations). The data is heavily skewed towards increased yield which elegantly demonstrates the general beneficial nature of M-trophs and highlights NewLeaf's focused strategy. In addition to yield increase, the best performing M-trophs enhanced early stand counts.

Phenotype x Genotype associations were analyzed using the PBK bioinformatics tool. The first step in identifying genes associated with desirable phenotypes (in this case yield increase >5%), and traits of interest, is to conduct a pan genome analysis. Here, using M-troph isolates, genes are enumerated across the entire collection. Some genes are present in only a single strain isolate, while some genes are present in all isolates. This pattern of gene presence and absence is used to perform a pangenome-wide association analysis. Associations are calculated between a specified phenotype (in this case yield increase >5%) or trait classification, and the pangenome-wide accessory genome (the set of genes present in only a subset of the isolates). With these phenotype and genotype data inputs, PBK can report a list of trait-associated genes along with their strength of association to the specified phenotype. NewLeaf's singular focus on M-trophs facilitates effective comparative and predictive genomics. Several potential marker genes associated with yield increase phenotype were identified and those markers will guide the M-troph strain nominations for 2018 soy bean seed treatment field trials.

## Bio-Complements and Ag Biologicals

Ag biologicals constitute 5% of total Ag input sales and usage is growing at an estimated average of 14% annually.^32^ Factors driving the increased use of Ag biologicals include:

▪ Grower and consumer demand for sustainable farming and plant-based proteins;
▪ High cost and extended time frame to bring new chemistries and GM traits to market; and
▪ Improvements in efficacy and consistency of Ag biological products

Second-generation Ag biologicals are designed as bio-complements to current products. Bayer CropScience's Poncho/Votivo 2.0 is an example of this product type. It is a seed treatment for corn, soybean, cotton and other crops that includes Poncho, a neonicotinoid insecticide (clothianidin), together with Votivo, a *B. firmus* for control of nematodes, and a Bt registered as a biostimulant and optimized for nutrient acquisition. Xanthion by BASF also combines an effective crop protection chemical with an Ag biological. NewLeaf Symbiotics introduced two bio-complement products in 2018. Terrasym 401 is a downstream soybean seed treatment to facilitate nitrogen availability and nutrient uptake in combination with rhizobia. Terrasym 402 is designed to complement peanut and other legumes as an in furrow application, improving, stand, vigor, and yield.

NewLeaf Symbiotics' pipeline includes several products that consist of M-trophs designed to complement chemical seed treatments in furrow. These combinations are designed to optimize complementarity between the Ag biological and chemicals thereby increasing the effectiveness of both.

A growing awareness of the importance of the microbiome in plant and crop health, along with innovations currently taking place in the expanding Ag biologicals field, signify notable progress towards increasing agricultural sustainability and crop yield. Of special interest is the new class of M-troph-based products, which provide a previously untapped opportunity to mitigate pressing global food production challenges.
